# Genome-wide linkage analysis of longitudinal phenotypes using σ^2^_A _random effects (SSARs) fitted by Gibbs sampling

**DOI:** 10.1186/1471-2156-4-S1-S12

**Published:** 2003-12-31

**Authors:** Lyle J Palmer, Katrina J Scurrah, Martin Tobin, Sanjay R Patel, Juan C Celedon, Paul R Burton, Scott T Weiss

**Affiliations:** 1 Channing Laboratory, Brigham & Women's Hospital and Harvard Medical School, Boston, Massachusetts, USA; 2 Department of Epidemiology and Biostatistics, Case Western Reserve University, Cleveland, Ohio, USA; 3 Department of Epidemiology and Public Health and Institute of Genetics, University of Leicester, United Kingdom

## Abstract

The study of change in intermediate phenotypes over time is important in genetics. In this paper we explore a new approach to phenotype definition in the genetic analysis of longitudinal phenotypes. We utilized data from the longitudinal Framingham Heart Study Family Cohort to investigate the familial aggregation and evidence for linkage to change in systolic blood pressure (SBP) over time. We used Gibbs sampling to derive sigma-squared-A-random-effects (SSARs) for the longitudinal phenotype, and then used these as a new phenotype in subsequent genome-wide linkage analyses.

Additive genetic effects (σ^2^_A.time_) were estimated to account for ~9.2% of the variance in the rate of change of SBP with age, while additive genetic effects (σ^2^_A_) were estimated to account for ~43.9% of the variance in SBP at the mean age. The linkage results suggested that one or more major loci regulating change in SBP over time may localize to chromosomes 2, 3, 4, 6, 10, 11, 17, and 19. The results also suggested that one or more major loci regulating level of SBP may localize to chromosomes 3, 8, and 14.

Our results support a genetic component to both SBP and change in SBP with age, and are consistent with a complex, multifactorial susceptibility to the development of hypertension. The use of SSARs derived from quantitative traits as input to a conventional linkage analysis appears to be valuable in the linkage analysis of genetically complex traits. We have now demonstrated in this paper the use of SSARs in the context of longitudinal family data.

## Background

The choice of phenotype is critical for the success of gene discovery in complex human diseases. In the case of most common, complex diseases, however, the choice is far from simple. Diseases such as cardiovascular disease are characterized by heterogeneity and are associated with a large number of intermediate phenotypes, which are themselves under complex genetic control and which may change markedly over time. The study of change in these intermediate phenotypes over time is important, as genes for disease severity or effect modification may be as or more important than genes encoding disease risk *per se*.

In this paper we explore a new approach to phenotype definition in the genetic analysis of longitudinal phenotypes. The generalized linear mixed model (GLMM) [1] provides the unifying framework and Gibbs sampling [2,3] a means of fitting these models.

We utilized data from the longitudinal Framingham Heart Study Family Cohort to investigate the familial aggregation and evidence for linkage to change in systolic blood pressure (SBP) over time. Our primary aim was to assess evidence for genetic effects on the rate of increase of SBP with age, as well as genetic effects on SBP at any given time. Our secondary aim was to derive sigma-squared-A-random-effects (SSARs) for the longitudinal phenotype, and to use these as a new phenotype in subsequent genome-wide linkage analyses.

## Methods

### Data

We analyzed the longitudinal Framingham Heart Study Family Cohort data set, provided for the Genetic Analysis Workshop, which included 4692 individuals in all 330 pedigrees. The primary response variable of the GLMMs and linkage models reported in this paper was the SBP measured over time. Explanatory covariates for all models included: sex, age, height, weight, number of drinks per week, number of cigarettes per day. Sex (m = 1, f = 0) was analyzed as a binary covariate. Other variables were treated as continuous. All continuous covariates were centered at or close to their mean. In order to allow for a nonlinear increase in SBP with age, quadratic and cubic age terms were also included. Although the model is Bayesian, the effects of each covariate on the phenotype can also be assessed using a pseudo-likelihood approach.

The covariates drink (number of drinks per week) and CPD (number of cigarettes per day) were only measured at 11 and 18 time points, respectively, in the first cohort. However, the majority of individuals had only one or two distinct values for these covariates throughout the study, so each missing value of drink and CPD was replaced with the mean of the covariates for that individual in the two observations surrounding the missing observation. This enabled us to increase the amount of useful observations without excluding important covariates from the model. The phenotypic component of the model included 24,746 observations for 2860 individuals. These observations all had non-missing values for SBP, height, weight, age, drink, and CPD.

### Variance components model

The models fitted in this analysis are extensions of our previously described variance components models for extended pedigrees with normal, binary, or censored survival phenotypes [4,5]. The original models are GLMMs with components of variance due to additive polygenic effects (σ^2^_A_), common family environment (σ^2^_C_), and common sibling environment (σ^2^_Cs_), and since the phenotype in this analysis is assumed normally distributed, an error variance (σ^2^_E_). The complex familial correlation structure is modeled through the use of shared random effects for each variance component. The model for the j^th ^individual in the i^th ^pedigree can be represented as:

μ_ij _= β_0 _+ β^T^X_ij _+ A_ij _+ C_ij _+ Cs_ij_

y_ij _~ N(μ_ij_, σ^2^_E_),

where y_ij _is the observed phenotype, X_ij _is a matrix of covariates, A_ij _~ N(0, V_A_), C_ij _~ N(0, V_C_), Cs_ij _~ N(0, V_Cs_) and the random effects are sampled from appropriate multivariate normal distributions with variance-covariance matrices (V_A_, V_C_, and V_Cs_) parameterized by σ^2^_A_, σ^2^_C_, and σ^2^_Cs_, respectively, and structured as described in [5]. Individuals without phenotypic data are used in generating the random effects but not in the main model.

The extensions not only allow for repeated measures on individuals, but also use the extra information available to assess evidence for genetic effects on the rate of change of the outcome over time [6]. At the k^th ^observation for the j^th ^individual in the i^th ^pedigree, the model can be expressed as:

μ_ijk _= β_0 _+ β^T^X_ijk _+ A_ij _+ C_ij _+ Cs_ij _+ age_ijk _× (β_age _+ A.time_ij _+ C.time_ij _+ Cs.time_ij _+ E.time_ij_)

y_ijk _~ N(μ_ijk_, σ^2^_E_),

where y_ijk _is the observed phenotype, X_ijk _is a matrix of covariates, β_age _is the mean gradient of age (for all individuals), A_ij _~ N(0, σ^2^_A_), C_ij _~ N(0, σ^2^_C_), Cs_ij _~ N(0, σ^2^_Cs_), A.time_ij _~ N(0, σ^2^_A.time_), C.time_ij _~ N(0, σ^2^_C.time_), Cs.time_ij _~ N(0, σ^2^_Cs.time_), E.time_ij _~ N(0, σ^2^_E.time_).

In this model time was measured using the covariate age. The random effects for the gradient of age, A.time_ij_, C.time_ij_, Cs.time_ij_, and E.time_ij_, have a covariance structure equivalent to that described previously [5], with each variance component replaced by its counterpart for the gradient, so (for example) in this part of the model σ^2^_A _is represented by σ^2^_A.time_. However, the variance components for the intercepts (σ^2^_A_, σ^2^_C_, σ^2^_Cs_, and σ^2^_E_) are independent of the variance components for the corresponding gradients (σ^2^_A.time_, σ^2^_C.time_, σ^2^_Cs.time_, and σ^2^_E.time_), and the estimated random effects for each variance component for the intercept are different from the estimated random effects for each variance component for the gradient. All random effects are constant across observations on the same individual; they do not change over time. Just as the size of σ^2^_A _can indicate whether additive genetic effects influence the outcome, the size of σ^2^_A.time _can indicate whether additive genetic effects influence the rate of change of the outcome with time. The narrow-sense heritability (*h*^2^_N_) was defined as the ratio of variance due to additive genetic effects (σ^2^_A _or σ^2^_A.time_) to the total phenotypic variance in SBP or rate of change in SBP [7].

The models were fitted using Gibbs sampling in WinBUGS v1.3 [8]. Because WinBUGS uses a Bayesian formulation, prior distributions must be specified for all parameters of interest. Vague normal N(0, 1000) priors were specified for fixed regression coefficients. Vague Pareto(1, 0.001) priors were specified on the precisions (inverses of the variances) of all random effects; these were equivalent to specifying uniform priors on the corresponding variances. Models were run for 45,000 iterations after a burn-in of 5000 iterations.

### Blood pressure adjustment

The problem of how to model continuous SBP when some individuals are on blood pressure treatment is complex. However, since complex modelling of SBP was not the main aim of this particular analysis, we chose a simple method of adjustment based on known average treatment effects [9,10]. This involved adding a constant (10 mm Hg) to each phenotype where the individual was on treatment, to reflect the 'true' SBP that might have been observed if the individual had not been on treatment.

### Linkage analysis

As described previously, the random effects due to σ^2^_A _(A_ij_, the sigma-squared A residuals, or SSARs) can be used as a continuous phenotype in a linkage analysis [11,12]. The SSARs for the gradient (A.time_ij_, the sigma-squared A time residuals or SSATRs), may also be used in such a way. The estimated SSARs and SSATRs were used as continuous phenotypes in a multipoint variance components linkage analysis that was performed using SOLAR v1.7.4 [13]. All 395 markers were included. The most common approach undertaken in previous genetic analyses of longitudinal data has been to estimate a simple linear gradient in each subject and then use this as a phenotype. Therefore, in order to compare our SSAR methodology to such an approach, we also estimated a simple linear gradient in each subject and used this as an outcome into the SOLAR linkage analyses.

## Results

Results from fitting the variance components model in WinBUGS are shown in Table 1. Both means ('best estimates') and 95% credible intervals are presented for each parameter. We deliberately excluded certain intermediate phenotypes (such as cholesterol and BMI) from the model even though they were clearly related to SBP, because we did not want to model away their contribution to variation in SBP.

The size of σ^2^_A.time _(relative to the other variance components for the gradient) gave a *h*^2^_N _estimate of ~9.2%, suggesting that genes do influence the rate of change of SBP with age, but that nongenetic influences due to shared familial or sibling environment are more important. In contrast, the variance components estimates for the intercept suggest that additive genetic effects are the largest component of variance for SBP at the mean age (*h*^2^_N _~ 43.9%).

**Figure 1:**
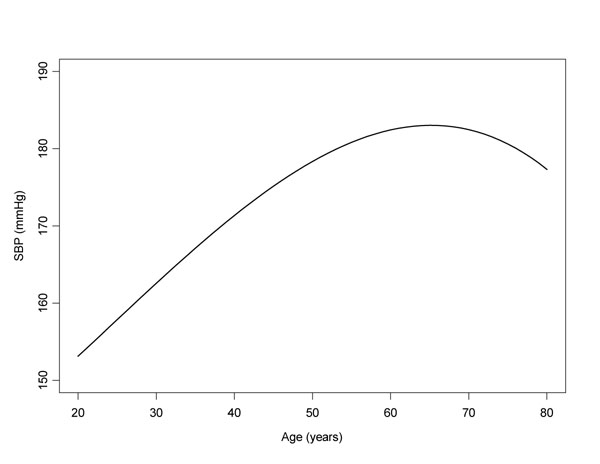
Modeled age-related change in SBP over time (model adjusted for treatment, weight, drink, sex and cohort membership).

The mean rate of change of SBP with each year was 0.62, suggesting that for the average individual, SBP increases by approximately 6.2 mm Hg every 10 years. A nonlinear effect was also apparent, since the credible intervals for both the quadratic and cubic terms excluded 0 (Figure 1).

Other covariates also appeared to influence SBP. Males tended to have lower SBP than females, and individuals from the second cohort tended to have lower SBP than those included in the first cohort. Both increasing weight and number of drinks consumed per day was significantly associated with increased SBP. Height and number of cigarettes per day appeared to show little association with SBP.

Results changed little when σ^2^_E.time _was excluded, when σ^2^_C _and σ^2^_C.time _were excluded, when σ^2^_Cs _and σ^2^_Cs.time _were excluded, and when the SBP adjustment (for those on blood pressure treatment) was changed to +5 mm Hg or +20 mm Hg (instead of +10 mm Hg). A number of genome-wide significant [14] linkages were found for both the SSARs and the SSATRs (Tables 2 and 3). No evidence of genome-wide significant linkage was found for the simple linear slope. The results suggested that one or more major loci regulating change in SBP over time may localize to chromosomes 2, 3, 4, 6, 10, 11, 17, and 19 (Table 2). The results also suggested that one or more major loci regulating level of SBP may localize to chromosomes 3, 8, and 14 (Table 3).

## Discussion

Our methods for the analysis of longitudinal data in pedigrees have several advantages. They make full use of the information contained in the repeated observations for each individual, without first requiring the information to be summarized in some way. It is simple to include extra covariates or single-nucleotide polymorphism genotypes (i.e., association analysis). They allow the inclusion of all pedigree members, without requiring decomposition of the pedigrees into smaller structures. These adjusted phenotypes can then be usefully used in linkage analyses. The results obtained suggest that our extended variance components models perform well in practice. They are relatively straightforward to fit, and produce useful and biologically plausible results.

Previous analyses of these data suggested a major locus regulating longitudinal SBP on chromosome 17 (multipoint LOD = 4.97 at 67 cM) [15]. This finding is broadly consistent (within 25 cM) of our finding on chromosome 17 for SSATR (multipoint LOD = 3.97 at 89 cM) (Table 2). Differences in results between this previous study [15] and our study may relate to differences in phenotypic definition (Levy et al. analyzed a residualized linear slope adjusted for BMI), differences in dealing with antihypertensive treatment, and differences in the sub-set of the Framingham Heart Study data analyzed.

Our results support a genetic component to both SBP and change in SBP with age, and are consistent with a complex, multifactorial susceptibility to the development of hypertension. The use of SSARs derived from quantitative traits as input to a conventional linkage analysis has previously been investigated using both real [11] and simulated data [12], and appears to be valuable in the linkage analysis of genetically complex traits. An analysis of the GAW13 simulated data using these methods [6] produced answers that corresponded well with the simulating model. We have now demonstrated in this paper the use of SSARs in the context of longitudinal family data.

## Figures and Tables

**Table 1:** Results from main model

**Parameter**	**Mean**	**95% Credible Interval**
Fixed effects		
β_0_	136.8	157.5 to 174.4
β.age	0.618	0.581 to 0.656
β.age^2A^	0.014	0.013 to 0.015
β.age^3B^	-1.884 × 10^-4^	-2.285 × 10^-4 ^to -1.493 × 10^-4^
β.cohort	-9.947	-11.02 to -8.916
β.male	-4.488	-5.597 to -3.375
β.wgt	0.516	0.485 to 0.547
β.wgt^2C^	-0.002	-0.003 to -0.001
β.hgt	-1.956	-8.216 to 4.211
β.drink	0.0453	0.034 to 0.057
β.cpd	-0.006	-0.030 to 0.016
Variance components		
σ^2^_A_	174.7	157.5 to 174.4
σ^2^_C_	45.46	30.44 to 62.0
σ^2^_Cs_	43.46	32.88 to 55.26
σ^2^_E_	134.2	131.5 to 136.9
σ^2^_A.time_	0.017	0.003 to 0.044
σ^2^_C.time_	0.031	0.015 to 0.049
σ^2^_Cs.time_	0.064	0.039 to 0.090
σ^2^_E.time_	0.072	0.047 to 0.099

^A^Quadratic term for age; ^B^Cubic term for age; ^C^Quadratic term for weight.

**Table 2:** Summary of linkage results to change in SBP (SSATR; LOD > 3)

**Chromosome**	**Phenotype**	**Marker**	**Location**	**LOD**
19	SSATR	GATA23B01	33	5.09
4	SSATR	GATA7D01	60	4.68
2	SSATR	GATA8F07	48	4.67
11	SSATR	GGAA5C04	58	4.59
10	SSATR	ATA31G11	28	4.26
6	SSATR	GGAA15B08	55	4.02
3	SSATR	GATA164B08	26	4.00
17	SSATR	ATA43A10	89	3.97

**Table 3:** Summary of linkage results to SBP (SSAR; LOD > 3)

**Chromosome**	**Phenotype**	**Marker**	**Location**	**LOD**
14	SSAR	GATA193A07	96	3.53
8	SSAR	GATA72C10	37	3.38
3	SSAR	GATA84B12	124	3.21
